# Utilization and perceptions of language assistance services by medical trainees: a pathway to language certification

**DOI:** 10.3934/publichealth.2024043

**Published:** 2024-07-03

**Authors:** Tucker Avra, Daniel Cordova, Breena Taira, Jesus R. Torres

**Affiliations:** 1 David Geffen School of Medicine at UCLA, Los Angeles, CA, USA; 2 Department of Emergency Medicine, Olive View-UCLA Medical Center, Sylmar, CA, USA; 3 Department of Emergency Medicine, David Geffen School of Medicine at UCLA, Los Angeles, CA, USA

**Keywords:** language services, interpreter access, language justice, health disparities, health equity, graduate medical education

## Abstract

**Background:**

Access to language assistance is a patient's right under federal law. Despite this, underuse of language services persists.

**Objective:**

The aim of this study was to explore the interest in obtaining bilingual certification and to describe perspectives on language services by resident physicians.

**Methods:**

Between May and August 2021, we conducted a cross-sectional survey of residents at a public, urban hospital serving mostly patients with limited English proficiency (LEP). We assessed resident perspectives on language services, exposure to language-related trainings, non-English language (NEL) skills, and interest in bilingual certification.

**Results:**

A total of 214 residents of 289 completed the survey (a 74% response rate). Of the 95 residents who used their NEL for patient care, 65 (68%) would be interested in bilingual certification. Sixty-nine (33%), 65 (31%), and 95 (45%) residents disagreed or strongly disagreed with being satisfied with the language services available, convenience, and sufficient equipment, respectively. Furthermore, 28 (13%) disagreed or strongly disagreed that they could achieve bi-directional communication with LEP patients.

**Conclusions:**

Over a quarter of the residents expressed interest in bilingual certification and were likely to pass the certification exam. Many reported using their own NEL skills without certification and held negative views on services and trainings.

## 1. Introduction

The dynamic demographics of the United States (US) pose unique challenges to the healthcare system. In particular, healthcare providers are increasingly tasked with providing care to a population that is both culturally and linguistically diverse. In the US, more than 60 million households speak a language other than English, and an estimated 42% of people in these households speak English less than “very well” [1]. Furthermore, during a thirteen-year period starting in 1990, patients with limited English proficiency (LEP) increased by 80% [2]. Healthcare systems are responsible for providing language assistance as required by law. Under Title VI of the 1964 Civil Rights Act and Section 1557 of the Affordable Care Act (ACA), access to language services is a patient right [3],[4]. Clear, bi-directional communication between the healthcare team and the patient is a critical component of the healthcare encounter, and anything short of this contributes to healthcare disparities [5].

Structural barriers to language-appropriate care in the healthcare setting compromise the delivery of high-quality care. For example, patients with LEP often experience more hospital admissions, a longer length of stay, and increased diagnostic testing. Additionally, patients with LEP report mistrust in the provider-patient relationship, decreased understanding of their diagnosis and/or treatment plan at discharge, and have more unplanned visits to the emergency department (ED) relative to patients proficient in English [6]–[10]. Furthermore, patients with LEP are often not represented in clinical research which may compound downstream health disparities [11]. The provision of appropriate language access (e.g., a professional interpreter and translation), and provider-patient language concordance has been shown to reduce these disparities and improve outcomes [5],[12],[13].

Despite the legal mandate and ample evidence of the positive effects of language services on patient-centered care, language services are routinely underutilized by healthcare providers. Reasons for this underutilization are multi-factorial, with time constraints and tradeoffs with efficiency routinely cited by medical residents [14]–[16]. Utilizing ad hoc interpreters, untrained hospital staff, or family members is common practice; however, these individuals may not always be able to adequately translate medical terms, be unable to maintain confidentiality, or may not be available [5],[7]. Healthcare providers may also rely on their own non-English language skills in lieu of professional language services to communicate with patients but may lack appropriate language competence or certification.

Guidance from the Health and Human Services' National Standards for Culturally and Linguistically Appropriate Services (CLAS) in Health and Health Care calls on healthcare organizations to ensure the competence of interpreters [17]. Additionally, under Section 1557 of the ACA, a “qualified” interpreter is someone whose skills have been tested in both English and the target language [4]. Certified bilingual healthcare providers are one component of the multifaceted approach to providing language services. Certification of residents improves language concordant care and thereby helps to close a disparity gap. In this study, we describe resident trainee perspectives on the utilization of language services and gauge interest in bilingual certification.

## 2. Materials and methods

### 2.1. Study design

The authors performed a cross-sectional, anonymous, online, 25-point questionnaire of resident physicians between May 2021 and August 2021. Trained research assistants recruited resident physicians via email, resident listservs, resident education conferences, intern orientation, and through resident mobile telephone text-messaging platforms. The survey took approximately 5–10 minutes to complete.

### 2.2. Setting

The study was undertaken at a large, public, urban, university-affiliated hospital with various residency programs. It is the only public hospital for the San Fernando Valley in northern Los Angeles County. More than 50% of the patient population prefer to receive their healthcare in a language other than English. The hospital subscribes to a remote interpreter service that provides spoken language assistance in 240 languages and is available around-the-clock. Telephone interpreters can be accessed from any telephone in the hospital, including via an app on the resident's mobile telephones. There are also video remote interpreter (VRI) devices available throughout the hospital and in-person interpreters, although with limited availability. Additionally, the institution has a system to test and certify bilingual employees and offers a monthly incentive of fifty dollars per pay period for those who pass the certification exam in any of the threshold languages in Los Angeles County (languages spoken by 5% or more of the population, including Armenian, Chinese, Farsi, Japanese, Korean, Spanish, Tagalog, Russian, and Vietnamese) and use the language in a patient-facing role on a continuous and frequent basis. In the study institution, resident physicians, however, are currently excluded from this language certification pathway.

### 2.3. Subjects

Residents who spend 50% or more of their training at the study institution were eligible for the study. This included residents in psychiatry, emergency medicine, OB/GYN, internal medicine, and primary care. Residents of all training years were considered eligible. This study was IRB approved by the Education and Research Institute at the Olive View-UCLA Medical Center prior to the commencement of any research.

### 2.4. Survey development

We began with a survey previously developed by our research group for ED providers and staff [18]. We then modified the questions to be appropriate to resident physicians of any specialty. We brought the draft questions to a multidisciplinary language access committee at our institution for comment, incorporated their suggestions, and had residents review them for face validity and understanding prior to deployment.

### 2.5. Measures and outcomes

Survey measures included the year in training, department, native language, and non-English languages (NEL) spoken. For those reporting NEL skills, we collected the frequency of NEL use with patients or clients and interest in bilingual certification. For those reporting NEL skills with interest in bilingual certification, we collected self-reported language ability as measured by the Interagency Language Roundtable (ILR) scale, a scale from level 0 to level 5 validated for the healthcare setting to assess self-reported language fluency, and the language spoken [19]. ILR levels zero through five indicate no proficiency, elementary proficiency, limited working proficiency, general professional proficiency, advanced professional proficiency, and functional native proficiency, respectively.

The primary outcome was the proportion of residents who would qualify for a proposed bilingual testing and certification pathway for the institution. Secondary outcomes included knowledge of and perspectives on language access including language services satisfaction, convenience of language services, sufficiency of language service equipment, sufficiency of best practices training, expected practices training received, knowledge on how to access written translations, knowledge of reporting procedures for language service difficulties, and confidence in achieving clear, bi-directional communication for patients with LEP. Each secondary outcome was measured on a 5-point Likert scale (strongly disagree, disagree, neutral, agree, strongly agree).

### 2.6. Analysis

Study data were collected using REDCap [20] (Research Electronic Data Capture) 20 hosted by the University of California Los Angeles, Clinical and Translational Institute (UCLA-CTSI). Data were analyzed using Stata 16.1 (StataCorp, College Station, TX). Descriptive statistics were used for the analysis.

## 3. Results

Of the 289 residents eligible for the survey, it was completed by 214, representing a 74% (214/289) response rate. For the 2021–2022 year, respondents were comprised of 69 (32%) incoming interns, 52 (24%) PGY1 residents, 41 (19%) PGY2, 33 (15%) PGY3, and 19 (9%) PGY4. Specialty representation included 60 (28%) in emergency medicine, 98 (46%) in internal medicine/primary care, 34 (16%) in OB/GYN, and 20 (9%) in psychiatry. The majority, 150 (76%), self-reported being able to speak a second language. The native languages of the resident respondents were 187 (88%) English, 14 (7%) Spanish, 3 (1%) Chinese (Cantonese or Mandarin), 3 (1%) Korean, and 6 (3%) other (see Table 1).

### 3.1. Language skills in hospital and resident certification

Although 88% reported English as their native language, a large proportion of residents reported speaking an additional language. NEL skills were reported by 150 (76%) residents.

Of these, 95 (64%) reported using their NEL skills with patients as part of their job, despite having no bilingual certification program for residents at the study institution. Furthermore, the usage of uncertified NEL skills to provide care to patients was common. When asked to describe the frequency of uncertified NEL use, respondents reported 11 (12%) rarely, 16 (17%) sometimes, 47 (50%) often, and 21 (22%) always. When asked if residents have employed their NEL to interpret for a patient or patient's family for whom they were not the primary provider, 72% responded often or always.

Of the 95 resident respondents who use NELs with patients as part of their job, 65 (68%) would be interested in being tested in their NEL to obtain a bilingual certification. Forty-seven (76%) residents would like to take the exam in Spanish, 1 (2%) in Arabic, 1 (2%) in Armenian, 5 (8%) in Chinese (Cantonese or Mandarin), 2 (3%) in Farsi, 3 (5%) in Korean, and 1 (2%) in Vietnamese. All of these languages are threshold languages for Los Angeles County. These 65 residents rated their proficiency in their non-English language based on the ILR as 3 (5%) Level 2, 25 (39%) Level 3, 19 (29%) Level 4, and 18 (28%) Level 5 (see Table 2).

When accounting for the threshold NEL spoken, interest in taking the bilingual exam and a self-reported ILR score of 3 or higher, in total 62 (27%) residents who responded to the survey would be both eligible and likely to pass a formal examination of their language skills to become certified.

**Table 1.** Participant demographics.

Variable	Frequency (%)	Cumulative Frequency
Resident Year (2021–2022)		
Incoming Intern	69 (32)	69
PGY1	52 (24)	121
PGY2	41 (19)	162
PGY3	33 (15)	195
PGY4	19 (9)	214
Department		
Emergency Medicine	60 (28)	60
Internal Medicine/Primary Care	98 (46)	158
OB/GYN	34 (16)	192
Psychiatry	20 (9)	212
Second Language		
No	47 (24)	47
Yes	150 (76)	197
Native Language		
English	187 (88)	187
Spanish	15 (7)	201
Chinese (Cantonese or Mandarin)	3 (1)	204
Korean	3 (1)	207
Other	5 (2)	213

**Table 2.** Language skills in hospital and resident certification.

Variable	Frequency (%)	Cumulative Frequency
*“Do you use your NEL skills with patients or clients of the DHS as part of your job?”*		
Yes	95 (64)	95
No	54 (36)	149
*“On average, how often do you use your NEL skills with patients or clients?”*		
Rarely	11 (12)	11
Sometimes	16 (17)	27
Often	47 (50)	74
Always	21 (22)	95
*“Would you be interested in being tested in your NEL to obtain a bilingual certificate from the County of Los Angeles?”*		
Yes	65 (68)	65
No	30 (32)	95
*“How would you rate your proficiency in your NEL?”*		
Level 1	0 (0)	0
Level 2	3 (5)	3
Level 3	25 (39)	28
Level 4	19 (29)	47
Level 5	18 (28)	65
*“What language would you want to take the exam in?”*		*Residents with an ILR Score of 3 or Higher (Frequency)*
Spanish	47 (76)	45
Chinese (Cantonese or Mandarin)	5 (8)	4
Korean	3 (5)	3
Other	5 (9)	5

### 3.2. Language services training

**Figure 1.**
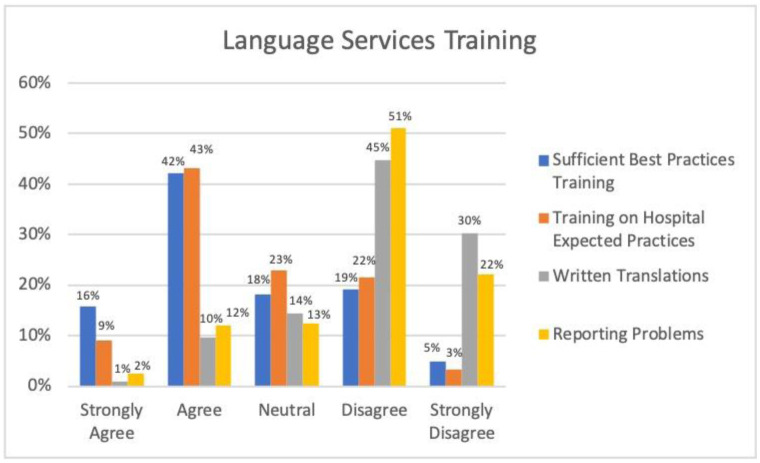
Language service training.

When asked if they received sufficient training on the best practices for working with an interpreter, 121 (58%) reported strongly agree or agree; however, 50 (24%) reported disagree or strongly disagree. Similarly, when asked if they received training on the expected practices on language access, a slight majority, 109 (52%), reported agree or strongly agree. When asked if they know how to access written translations, only 22 (11%) residents reported strongly agree or agree, with the vast majority, 188 (75%), reporting disagree or strongly disagree. Lastly, when asked if they know how to report a problem when experiencing difficulty in accessing language services, 152 (73%) reported disagree or strongly disagree (see Figure 1).

### 3.3. Perceptions of language services availability

**Figure 2.**
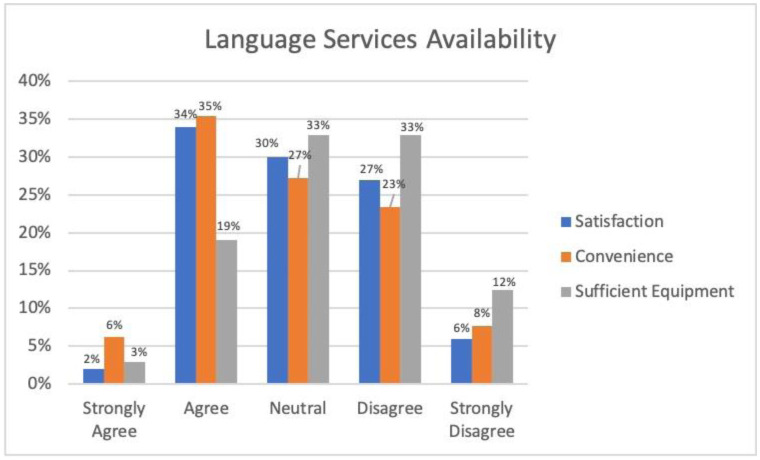
Perceptions of language service availability.

Residents were asked to rate their satisfaction, convenience, and perceptions about the availability of access to language services. When asked if they are satisfied with the language services offerings at the study institution, the majority, 141 (63%), reported strongly disagree, disagree, or neutral. Eighty-seven (41%) residents reported strongly agree or agree that accessing language services was a convenient process. Lastly, 46 (22%) respondents stated that they strongly agree or agree when asked if access to provided equipment was sufficient (see Figure 2). Despite these perceptions, 119 (57%) respondents stated that they strongly agree or agree that they can achieve clear, bi-directional communication with LEP patients.

## 4. Discussion

Patients with non-English language preferences have the right to appropriate language services under Title VI of the 1964 Civil Rights Act and Section 1557 of the ACA [3],[4]. However, despite the availability of language service resources in the clinical setting at the study institution, barriers to access persist. The majority of respondents reported that accessing language services was not convenient, they were not satisfied with the availability of language service offerings, and the equipment used for interpretation was not sufficient. This may present a barrier to accessing and utilizing adequate professional interpretation.

Written discharge instructions are important to convey the expected treatment and follow-up plan. Often, providers rely on non-professional machine translation services that were not designed to be employed in the healthcare setting (i.e., Google) to translate the written instructions. Studies show that utilization of such services may lead to inaccurate translations [21]. Even though written translation is available as part of the language services at the study institution, the vast majority of residents reported lack of familiarity with these services. Inaccurate and misinterpreted discharge instructions from machine translation may result in harmful outcomes [22]. Although available, the practicality of employing professional written translation in the clinical setting for discharge instructions was not assessed in this study.

In this study, the vast majority (76%) of respondents reporting having NEL skills in addition to English. Although the study institution excludes resident physicians from its bilingual certification program, nearly two-thirds of those that reported NEL skills use these skills to provide patient care, with 72% using their NEL often or always. Diamond et al. discovered that residents use their own NEL skills and underuse professional interpreters because of ease, despite the knowledge that it may have implications for healthcare quality [14]. Although the reasons were not directly ascertained, a large percentage of residents in this study also use their own NEL skills, potentially impacting patient care. Furthermore, these individuals also use their NEL to provide language assistance to other provider's patients, with 72% reporting this use often or always. This is consistent with other studies that show that providers employ non-certified staff, other providers, and/or family members, which may pose a threat to patient care [23]. Although common practice in the clinical setting, the utilization of non-certified individuals, including other residents, to aid in the interpretive process violates federal regulations and is a patient safety issue, given that the NEL skills may not be sufficient to achieve clear, bi-directional communication. This additional use of NEL skills for other providers adds an additional, uncompensated strain to the workload of the residents.

Section 1557 of the ACA explicitly defines a “qualified bilingual/multilingual” staff member as one that is “1) Proficient in speaking and understanding both English and at least one other spoken language, including any necessary specialized vocabulary, terminology and phraseology; and 2) Able to effectively, accurately, and impartially communicate directly with limited English proficient individuals in their primary languages” [4]. In our sample, 65 residents out of 95 reported using their NEL skills for patient care, with 27% self-rating their NEL ability at 3 or above in the IRL scale. This provides a significant opportunity to boost the number of qualified bilingual/multilingual staff as is demonstrated in other studies [24]. Certifying residents provides an avenue for health equity by ensuring that patients receive adequate access as afforded by federal regulation in addition to language concordant care, improving patient safety and health outcomes [5].

### 4.1. Limitations

This study has several limitations as it is based on a survey that was conducted at a single, urban institution that serves a large population of LEP. Although we achieved a high response rate, we only surveyed those residents that spent 50% of more of their time at the study institution, potentially not fully representing our residents' abilities or behaviors in employing professional interpreters. Furthermore, since our institution serves a majority LEP patient population, there may be more opportunities that the institutional culture may normalize using one's uncertified NEL skills to a greater extent than other locations with fewer LEP patients. Since our results represent the experiences at a single institution, our results may not be generalizable. However, this is one of the first studies evaluating NEL skills and interest in certification in a cross-disciplinary population of residents.

## 5. Conclusions

The majority of residents with NEL expressed interest in taking a certification examination, and a large proportion of residents reported using their own NEL skills despite lacking a formalized certification pathway at the study institution. Residents reported encountering various challenges in utilizing language access offerings. They faced difficulties in knowing how to access written translation, how to report issues related to the language services, and had unfavorable views regarding the accessibility, convenience, and equipment used to facilitate services. These findings will be presented to the chief medical officer and designated institutional official at our institution, and used to advocate for a pilot project for resident certification, eventually providing a bilingual certification pathway for residents across the hospital and county.

## Use of AI tools declaration

The authors declare they have not used artificial intelligence (AI) tools in the creation of this article.
